# Heterogeneous development of methanogens and the correlation with bacteria in the rumen and cecum of sika deer (*Cervus nippon*) during early life suggest different ecology relevance

**DOI:** 10.1186/s12866-019-1504-9

**Published:** 2019-06-11

**Authors:** Zhipeng Li, Xiaoxu Wang, Ting Zhang, Huazhe Si, Chao Xu, André-Denis G. Wright, Guangyu Li

**Affiliations:** 1 Department of Special Animal Nutrition and Feed Science, Institute of Special Animal and Plant Sciences, Chinese Academy of Agricultural Sciences, Changchun, China; 2 College of Agricultural, Human, and Natural Resource Sciences, Washington State University, Pullman, Washington USA

**Keywords:** Gastrointestinal tract, Microbiome succession, Bacteria, Methanogens, Regional difference

## Abstract

**Background:**

Enteric methane from the ruminant livestock is a significant source in global greenhouse gas emissions, which is mainly generated by the methanogens inhabiting the rumen and cecum. Sika deer (*Cervus nippon*) not only produces less methane than bovine, but they also harbor a distinct methanogen community. Whereas, knowledge of methanogens colonization in the rumen and cecum of sika deer is relatively still unknown, which could provide more insights to the manipulation of gut microbiota during early life.

**Results:**

Here, we examined the development of bacteria and methanogens in the rumen and cecum of juvenile sika deer from birth to post-weaning (1 day, 42 days and 70 days, respectively) based on next generation sequencing. The results showed that the facultative anaerobic bacteria were decreased and the cellulolytic bacteria were increased. However, methanogens established soon after birth thrived through the whole developmental period, indicating a different succession process than bacteria in the GIT, and the limited role of age and dietary change on GIT methanogens. We also found *Methanobrevibacter* spp. (Mean relative abundance = 44.2%) and *Methanocorpusculum* spp. (Mean relative abundance = 57.5%) were dominated in the rumen and cecum, respectively. The methanogens also formed specific correlations with bacteria under different niches, suggesting a role of ecology niche on methanogen community.

**Conclusions:**

This study contributes to our knowledge about the microbial succession in GIT of sika deer, that may facilitate the development of targeted strategies to improve GIT function of sika deer.

**Electronic supplementary material:**

The online version of this article (10.1186/s12866-019-1504-9) contains supplementary material, which is available to authorized users.

## Background

Enteric methane from the ruminant livestock is a significant source in global greenhouse gas emissions [1–3]. The half-life time of methane in the atmosphere is 12.4 years [4], which also has an atmospheric warming potential 25 times higher than carbon dioxide [5]. Enteric methane is produced by the methanogenic archaea distributed in the gastrointestinal tract (GIT), which uses C_1_ and C_2_ carbon sources to dispose the hydrogen in the last step of the anaerobic chain [6]. Methanogenesis is important to prevent the accumulation of reducing equivalents and the overall inhibition of rumen fermentation [7, 8], but also leads to 2–12% dietary energy loss to the host [9]. However, the effects of the presently applied approaches (e.g., dietary supplement and microbiota transplant) on adult ruminants is limited or short [10, 11], due to the resistance and recovery characteristics of GIT microbiota [12]. On the other hand, it is known that the microbial community in the GIT is gradually colonized by specific microorganisms [13, 14]. Therefore, understanding how methanogens establish in the GIT is crucial to develop successful approaches or methods to manipulate the microbiota development.

Host genetics was one of the most important factors affecting the methanogen community and the associated methane emissions in the bovine rumen [15, 16]. Moreover, a recent study demonstrated that deer produced less methane compared to cattle [17]. Na et al. (2017) revealed that methane per unit was lower in sika deer (*Cervus nippon*) than goat [18]. Accordingly, Henderson et al. (2015) demonstrated that the methanogen community in the rumen of Cervids was indeed different from that in the Bovinae [19]. These results suggested that exploring the methanogen development in the GIT of sika deer could provide more insights into the establishment of methanogen community, and the manipulation of ruminant in early life. However, there has not been report on the methanogens development in GIT of sika deer.

Although the rumen is the main resource of enteric methane, the cecum also provides up to 8.6% of metabolizable energy to host [20], and also generates additional methane production (∼10% of the ruminant methane) [21]. Thus examining methanogens in the rumen and cecum at different time during early life could provide a comprehensive picture of methanogen development. Up to now, previous studies of methanogens in the gut of pre-weaned ruminants revealed that *Methanobrevibacter* spp. were the dominated methanogens in rumen/or feces of calves and goat before weaning [13, 22–25].

There are significant differences in physiology and function between the rumen and cecum. First, the rumen is an efficient site to degrade plant materials with the production of large amounts of hydrogen, while the cecum mainly ferments un-degraded carbohydrates (structural carbohydrates) that bypass the rumen. Second, the bacterial communities in the rumen and cecum are significantly different, which produce substrates for the methanogens growth [26]. Third, the substrate preference and the adaptation to oxidative environments for different methanogen species are varied [27, 28]. Last, there is a lack of protozoa in the cecum as compared to the rumen [29]. These facts contribute to the hypothesis that the first methanogens colonized in the rumen and cecum are different.

Therefore, the present study aimed to describe and compare methanogens in the rumen and cecum, and bacteria in the rumen of sika deer at 1 day, 42 days and 70 days (10 days after weaning), respectively; and to explore the correlation between bacteria and methanogens in the rumen and cecum ecosystems.

## Results

### Development of the bacterial community in rumen

**Fig. 1:**
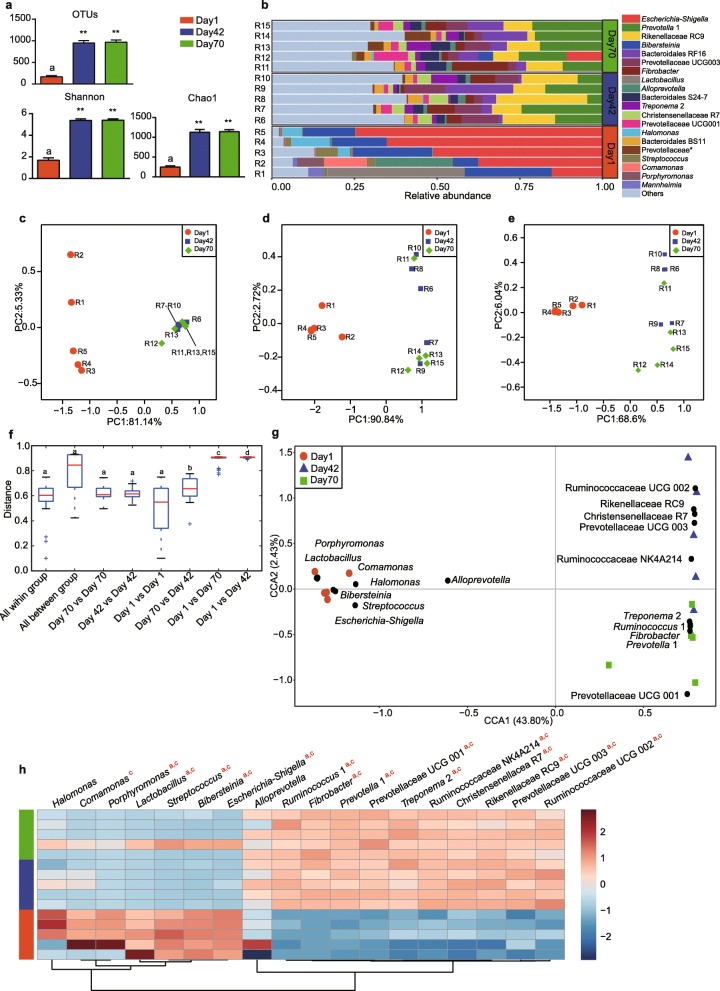
The bacteria community composition in the rumen across three time points. **a** Diversity and richness indices at days 1, 42 and 70. ** *p* < 0.01. **b** Bacterial composition at the genus level in the rumen. The asterisk means the unclassified bacteria at the family level. Principal coordinate analysis (PCoA) of rumen microbiota based on unweighted unifrac (**c**), weighted unifrac distance (**d**) and bray-curtis distance (**e**). Box plots showing within-group similarity and between-group dissimilarity based on bray-curtis distance (**f**). The different letters (a, b, c, d) means significant differences (Kruskal-Wallis tests, FDR-adjusted q < 0.05). Canonical correlation analysis (**g**) and heat-map (**h**) showing the significant bacteria taxon in the rumen across three time points. Black circles indicate the representative taxon at each time points. The letters a, b, and c indicate significant differences between day 1 and day 42, between day 42 and day 70, and between day 42 and day 70, respectively. R = Rumen

A total of 369,136 sequences were obtained in the present study, which were classified into 1858 operational taxonomic units (OTUs) after sub sample. The OTU numbers and Shannon and Chao1 indices significantly increased from day 1 to days 42 and 70 (*p* < 0.05) (Fig. 1a).

Based on these OTUs, we identified a total of 20 phyla in the rumen across three time points (Additional file 1: Figure S1), which were further classified into 340 genera (Fig. 1b). *Escherichia-Shigella* (48.8%) was the most dominant bacteria at day 1, followed by *Bibersteinia* (18.0%), *Lactobacillus* (8.5%), *Alloprevotella* (4.8%), and *Halomonas* (4.2%), accounting for 84.3% of the overall bacterial composition. At day 42, bacteria belonging to *Rikenellaceae* RC9 (15.8%) were predominant, followed by *Prevotella* 1 (13.6%), *Prevotellaceae* UCG 003 (8.3%), *Bacteroidales* RF16 (6.0%), and *Fibrobacter* (4.8%). These bacteria accounted for 48.5% of the bacterial community. At day 70, *Prevotella* 1 bacteria (17.4%) were the most abundant genus, followed by *Rikenellaceae* RC9 (8.0%), then *Bacteroidales* RF16 (7.7%), *Fibrobacter* (6.1%), *Bacteroidales* S24–7 (4.6%), *Prevotellaceae* UCG 003 (4.5%), and *Prevotellaceae* UCG 001 (4.2%). These bacteria which made up 52.3% of the bacterial composition.

The principal coordinate analysis (PCoA) results showed that the bacterial community composition in the rumen at day 1 was clearly distinct from that at days 42 and 70 based on unweighted unifrac distance, weighted unifrac distance, and bray-curtis distance (Fig. 1c-e), explaining at least 68.6% of the variation. Comparison of the bacterial community distance also revealed significant differences between day1 and days 42 and 70 (Fig. 1f).

Canonical correlation analysis (CCA) identified a total of 18 bacterial taxa across three time points (Fig. 1g). The abundances of *Porphyromonas*, *Lactobacillus*, *Streptococcus*, *Bibersteinia*, and *Escherichia-Shigella* were significantly decreased at days 42 and 70 compared to those at day 1 (*p* < 0.05, Fig. 1h). On the contrary, the abundance of *Ruminococcus* 1, *Fibrobacter*, *Prevotella* 1, *Prevotellaceae* UCG 001, *Treponema* 2, *Ruminococcaceae* NK4A214, *Christensenellaceae* R7, *Rikenellaceae* RC9, *Prevotellaceae* UCG 003 and *Ruminococcaceae* UCG 002 were significantly increased at days 42 and 70 as compared to day 1 (*p* < 0.05, Fig. 1h).

### Sequencing summary for rumen and cecum methanogens at days 1, 42 and 70

**Fig. 2:**
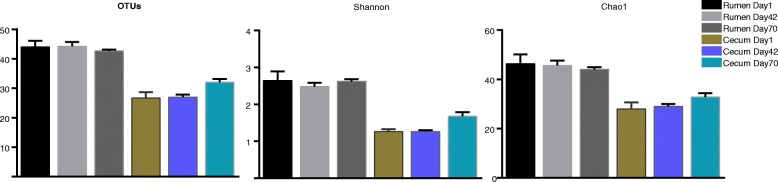
Diversity and richness indices in the rumen and cecum of sika deer from birth to after weaning

In the present study, a total of 723,689 methanogen 16S rRNA gene sequences were obtained from 13 animal samples (two animals failed to produce large sequences), with 340,032 sequences from the rumen (19,412 to 33,755 for each sample), and 369,458 sequences from the cecum (14,199 to 37,783 for each sample), respectively. These sequences were classified into 56 OTUs based on 97% sequence similarity. The OTU numbers and Shannon and Chao1 indices in both the rumen and cecum were not significantly different across the three time points (*p* > 0.05). However, the diversity and richness indices in the rumen was higher than that of the cecum (Fig. 2).

### Methanogen composition in rumen and cecum with the development

**Fig. 3:**
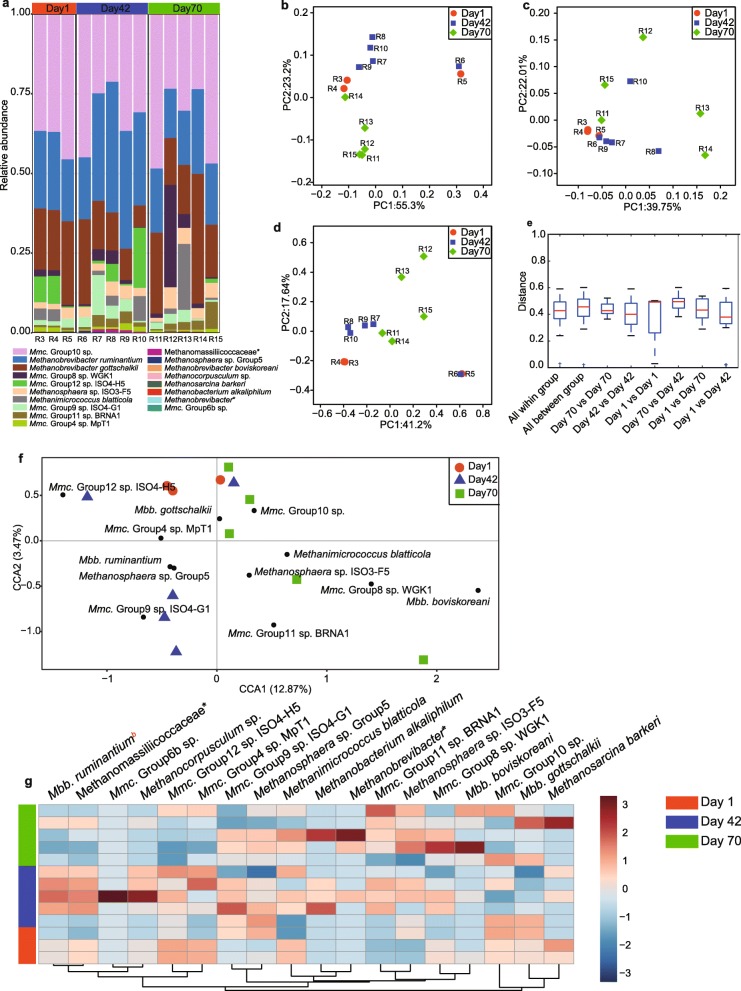
The methanogen community structure and composition in the rumen of sika deer at three time points. **a** The methanogen composition at species level in the rumen at days 1, 42 and 70. PCoA results based on unweighted unifrac distance (**b**), weighted distance (**c**) and bray-curtis distance (**d**), and the within-, and between- group distance based on bray-curtis distance (**e**). Canonical correlation analysis (**f**) and heat-map (**g**) showing the significant methanogens in the rumen across three time points. Black circles indicate the representative taxon at each time points. The letter b indicates significant differences between day 42 and day 70. *Mmc*. = *Methanomassiliicoccaceae*; *Mbb*. = *Methanobrevibacter*. The asterisk means the unclassified methanogens at the family or genus level. R = Rumen

In the rumen, a total of 18 species were identified based on the 53 OTUs at three time points (Fig. 3a). The *Methanomassiliicoccaceae*-affiliated group, *Mmc*. Group10 sp. (Day1: 39.7%; Day42: 31.7%; Day70: 34.5%), *Methanobrevibacter ruminantium* (Day1: 22.8%; Day42: 32.1%; Day70: 19.6%), and *Methanobrevibacter gottschalkii* (Day1: 21.4%; Day42: 14.1%; Day70: 22.6%) were the dominant methanogens in the rumen across three time points. PCoA results showed that the methanogen community in the rumen was not significantly separated at the three time points based on unweighted unifrac distance, weighted unifrac distance, and bray-curtis distance (Fig. 3b-d). Moreover, the methanogen community distances at days 1, 42 and 70 were also not significantly different (Fig. 3e).

We also applied CCA to identify the methanogens species representing each time point. Although a total of 12 methanogens species in the rumen were identified to be associated with the three different time points (Fig. 3f), only the abundance of *Mbb. ruminantium* was significantly different between days 42 and 70 (Fig. 3g). The abundance of *Mmc*. Group 12 sp. ISO4-H5 was much higher at days 1 (5.7%) and 42 (5.2%) than that at day 70 (0.3%), while the proportion of *Mmc*. Group 8 sp.WGK1 was more abundant at day 70 (7.9%) than that at days 1 (1.4%) and 42 (1.9%).

**Fig. 4:**
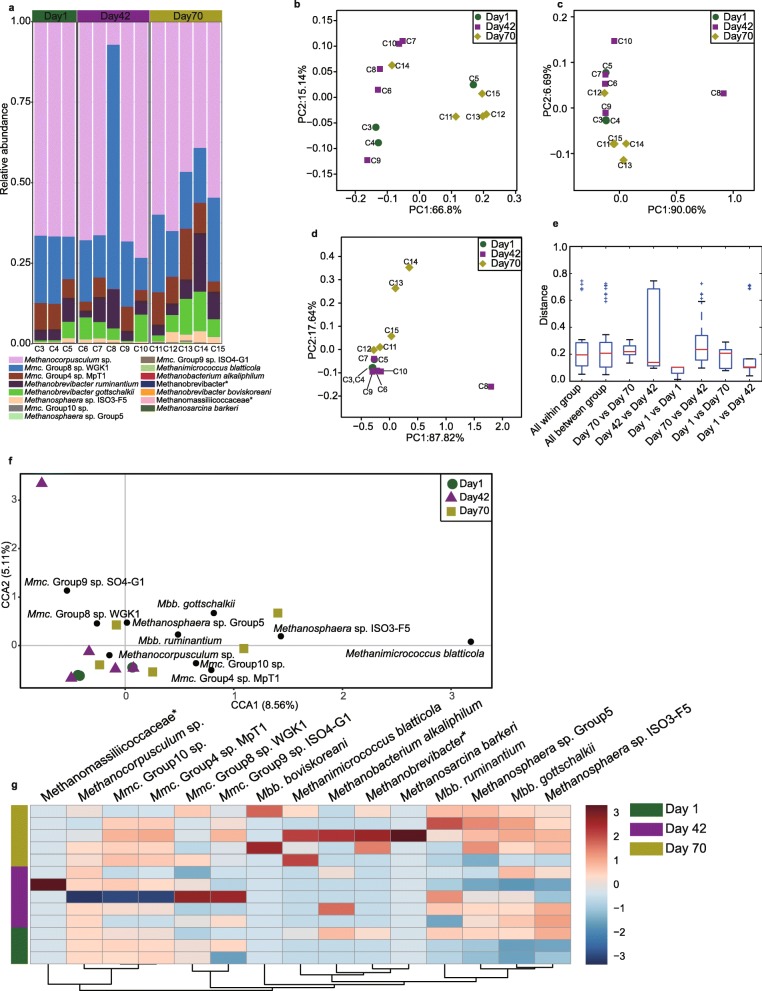
The methanogen community composition in the cecum of sika deer at three time points. **a** The methanogen composition at species level in the cecum at days 1, 42 and 70. PCoA results based on unweighted unifrac distance (**b**), weighted distance (**c**) and bray-curtis distance (**d**), and the within-, and between- group distance based on bray-curtis distance (**e**), showing the variation of methanogen community at three time points. Canonical correlation analysis (**f**) and heat-map (**g**) showing the significant methanogens in the rumen across three time points. Black circles indicate the representative taxon at each time points. *Mmc*. = *Methanomassiliicoccaceae*; *Mbb*. = *Methanobrevibacter*. The asterisk means the unclassified methanogens at the family or genus level. C = Cecum

In the cecum, a total of 15 methanogens species were identified based on the 42 OTUs at three time points (Fig. 4a). The top 3 methanogens species were *Methanocorpusculum* spp. (Day1: 66.6%; Day42: 56.6%; Day70: 53.1%), *Mmc*. Group 8 sp. WGK1 (Day1: 18.3%; Day42: 27.6%; Day70: 19.8%), *Mmc*. Group4 sp. MpT1 (Day1: 7.3%; Day42: 4.0%; Day70: 9.4%), accounting for more than 82% of the overall methanogens composition.

PCoA results revealed that the cecum methanogen community at three time points was not significantly different (Fig. 4b-e). The representative methanogens at three time points in the cecum of sika deer were identified based on CCA, resulting in the identification of a total of 10 methanogen species (Fig. 4f). However, these methanogen species were not significantly different (Fig. 4g). The relative abundance of *Mbb. ruminantium* (Day1: 4.7%; Day42: 5.9%; Day70: 7.9%) and *Mbb. gottschalkii* (Day1: 2.0%; Day42: 4.8%; Day70: 7.2%) tended to increase during the development.

### Correlation between methanogens and bacteria in rumen and cecum

**Fig. 5:**
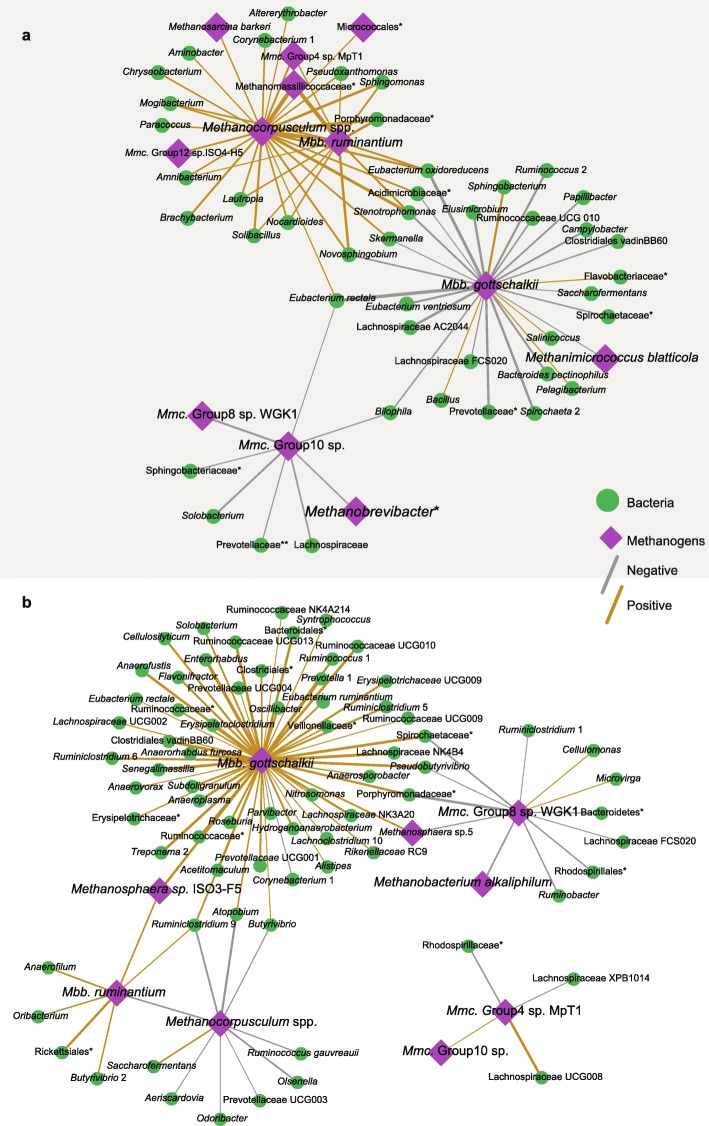
The co-occurrence correlation between methanogens (species level) and bacteria (genus level) in rumen (**a**) and cecum (**b**). The co-occurrence was constructed from the taxon abundance across the tree time points for rumen and cecum, respectively. The gray and gold lines represent the negative and positive correlation, respectively. The width of lines indicates the correlation coefficient, with bold lines for a greater coefficient, while the thick lines for a weaker coefficient. The pink rhombus and green circle represent the methanogens and bacteria, respectively. The asterisk means the unclassified bacteria genera or methanogens species

In the rumen (Fig. 5a), the methanogens *Methanocorpusculum* spp. and *Mbb. ruminantium* positively were correlated with 37 taxa, including *Mmc*. Group4 sp. MpT1 and the unclassified Methanomassiliicoccaceae and *Eubacterium oxidoreducens*. *Mbb. gottschalkii* negatively was correlated with a total of 22 taxa, including *Eubacterium oxidoreducens*, *Eubacterium rectale*, *Eubacterium ventriosum*, *Ruminococcus* 2 and *Ruminococcaceae* UCG 010. The dominated methanogens *Mmc*. Group10 sp. also negatively correlated with *Mmc*. Group8 sp. WGK1, the unclassified *Methanobrevibacter*, the unclassified *Lachnospiraceae*, and the unclassified *Prevotellaceae*.

In the cecum (Fig. 5b), *Mbb. gottschalkii* formed the mostly positive correlation with 45 taxa. *Mmc*. Group8 sp. WGK1 positively correlated with *Cellulomonas*, and negatively correlated with *Ruminiclostridium* 1, Lachnospiraceae FCS020, *Ruminobacter*, the unclassified *Spirochaetaceae*, *Pseudobutyrivibrio*, and the unclassified *Porphyromonadaceae*. *Methanocorpusculum* spp. negatively correlated with *Ruminiclostridium* 9, *Butyrivibrio*, *Ruminococcus gauvreauii*, and *Prevotellaceae* UCG003. *Methanomassiliicoccaceae* Group4 sp. MpT1 positively correlated with *Mmc*. Group10 sp. and *Lachnospiraceae* UCG008, but negatively correlated with *Lachnospiraceae* XPB1014 and the unclassified *Rhodospirillaceae*.

## Discussion

### Age related succession pattern of rumen bacteria

The bacterial diversity, community composition at day 1 was significantly different from that at days 42 and 70 (Fig. 1a and c-f), consistent with the bacterial development in previous studies about rumen [14, 22, 30, 31]. These results suggested that age was a factor to influence the bacteria colonization in GIT. Moreover, we found the *Firmicutes*/*Bacteroidetes* ratio was also decreased from day 1 (4.6) to days 42 (0.5) and 70 (0.4) (Additional file 1: Figure S1), consistent with the observation in the rumen microbiome of Indian Kankrej cattle that showed an increase of phylum *Bacteroidetes* when the diet contained much more concentrate [32]. These results suggested that age confounded with the dietary changes affected rumen bacterial colonization [33]. However, the abundant bacteria including *Escherichia-Shigella* (48.8%), *Bibersteinia* (18.0%), and *Lactobacillus* (8.5%) at day 1 were different from the previous reports on the rumens of calves [14, 34], lambs [30] and goats [35], suggesting the variation of the established rumen bacteria after birth. This may be related to the milk composition, animal species, and environment [10, 36]. *Lactobacillus* spp. and *Escherichia* spp. are facultative anaerobic bacteria, which could create the anaerobic conditions that allow for the establishment and succession of obligate anaerobes in gut [37]. These findings suggested that the dominated bacteria in rumen after birth contributed to the colonization and activities of the obligate anaerobes. Along with the increased age, the prevalent bacteria including *Rikenellaceae* RC9 (15.8%), *Prevotella* 1 (13.6%), *Prevotellaceae* UCG 003 (8.3%), and *Fibrobacter* (4.8%), and species within *Ruminococcaceae* family were significantly increased (Fig. 1g-h), which all played important roles in the initiation of the breakdown of plant fiber in rumen microbial fermentation [38]. *Prevotella* spp. have the capability to utilize starches, simple sugars, and other non-cellulosic polysaccharides as energy [39]. The genera *Ruminococcus* and *Fibrobacter* were the two major cellulolytic genera frequently found in the adult rumen [38, 40, 41]. However, the difference between day 42 and day 70 were not significant. Similarly, a previous study documented that the rumen of pre-ruminant calves has maintained a stable function and metabolic potentials [42]. These results indicated that metabolic ecology of the rumen may be established before the weaning.

### Methanogens colonization in rumen and cecum is not associated with age

Diversity and richness indices that were not significantly different with the development in the rumen and cecum (Fig. 2), were also observed in rumen solid and liquid fractions [13, 31, 43]. PCoA results demonstrated that the methanogen community membership and structure were also not significantly different (Figs. 3b-g and 4b-g), and some minor methanogens were significantly changed (Figs. 3f-g and 4f-g), which was consistent with previous findings in the rumen of lambs, goats and dairy calves [13, 24, 25, 31, 43]. These results suggested that age was not a key factor affecting methanogen colonization after the birth to after weaning. The reason for the insignificant influence of age on methanogen establishment in both the rumen and cecum was not clear, which may be related to the dietary composition [33]. However, previous results demonstrated that the dam may affect methanogen colonization [43], and the changes in the composition and abundance of methanogen communities were attributed exclusively to the substrate in the rumen, these results suggested the possible role of ecological environment on rumen methanogens colonization. However, the present study was limited to a short term time, and failed to examine the difference between adult and young animals.

### Regional difference of methanogens between rumen and cecum after birth

The results showed that the composition of the methanogen community in the rumen were significantly different from that in the cecum regardless of time (Additional file 2: Figure S2, Figs. 3a and 4a), indicating that the upper and lower GIT maybe a critical component in affecting the methanogen community. This discrepancy was likely to result from several different characteristics between the rumen and cecum. The rumen receives a large volume of saliva, which buffers the acidity from volatile fatty acids [44], while the cecum receives no saliva. Within the rumen more than 90% of dietary plant cell walls [45] and 20–90% of the starch are degraded [46], while nutrients entering the cecum were comprised of recalcitrant carbohydrates. The luminal content passage rate between the rumen and cecum, and the microbial composition were also different [47], such as the presence of reductive acetogenesis and the low number or absence of hydrogen-producing protozoa in the cecum [29].

The predominant methanogens in the rumen were *Methanobrevibacter* spp. and *Mmc*. Group10 sp. (Fig. 3a), which are agreement with the previous findings in the rumen of lambs [43], goats [13], calves [22, 23, 31], and adult ruminants [19]. However, *Methanocorpusculum* spp. were much more abundant in the cecum (Fig. 4a), in contrast to the prevalence of *Methanobrevibacter* spp. in cecal contents of the growing lambs [47], calves [23, 48], adult reindeer [29] and roe deer [26]. Together, these results demonstrated that the dominant methanogens in the rumen were universally distributed, which may not be affected by host genetics, diet and age, while cecum methanogen composition may be affected by both host genetics and diet composition. Surprisingly, the dominant *Methanocorpusculum* species in the cecum of sika deer were abundantly found in the gut of termite [49], and the hindgut of captive white rhinoceroses [50]. The proportion of *Methanocorpusculum* spp. was increased in horse feces when forage was fed to horse [51]. Although both *Methanobrevibacter* spp. and *Methanocorpusculum* spp. utilize hydrogen for methanogenesis [52], they were classified into class I and class II based on 16S rRNA sequences, respectively, reflecting metabolic differences [53]. Recent studies also documented that the two taxonomic classes of methanogens exhibited different tolerance to oxygen [27], and shifted the metabolism to energy conservation based on the substrate [28]. We further blasted the representative sequences of *Methanocorpusculum* spp. (2 OTUs) at NCBI database, and found that they showed the highest identity with *Methanocorpusculum labreanum* (98%), which may use the membrance-bound hydrogenase Mbh or energy-converting hydrogenase Ech to couple heterodisulfide reduction to a transmembrane ion gradient [52]. These results suggested *Methanocorpusculum* spp. may have adapted the environmental ecology of cecum (e.g., pH and host receptors), and played a possibly role of contributing to hindgut fermentation of carbohydrates. On the other hand, the *Methanomassiliicoccaceae*-related species (*Mmc*. Group10 sp.) were abundant in the rumen that usually utilized methanol or methyl compounds as substrate for growth [54, 55]. A recent study demonstrated that the utilization of methyl compounds among various lineages of *Methanomassiliicoccaceae* was different [56]. Borrel et al. (2017) found these sequences along with Candidatus *Methanomassiliicoccus intestinalis* Mx1 could utilize the trimethylamine, and that *Escherichia* spp. were important players in trimethylamine production from choline and L-carnitine [56], which were also dominated in the rumen of sika deer at day 1. Therefore, these results suggest that the ecological niche and the possible existence of opportunistic associations between rumen methanogens and bacteria [31] affected methanogen seeding.

### Correlation between methanogens and bacteria in rumen and cecum

To further explore the possibly different ecological niche for methanogen colonization resulting from bacteria, we looked for a correlation between methanogens and bacteria in the rumen and cecum [57], respectively. The results showed there were no strong correlations between the most abundant bacteria and methanogens in both rumen and cecum (Fig. 5), consistent with the previous findings [19]. However, there were distinct correlations between some less abundant bacteria and methanogens in the rumen and cecum, respectively. In the rumen, *Mbb. ruminantium* and *Mbb. gottschalkii* positively and negatively correlated with the different species belonging to the genus *Eubacterium* (Fig. 5a). These bacteria mainly produced butyrate [58], indicating the possible role of butyrate on methanogens proliferation of *Methanobrevibacter* spp. in rumen through the consumption of fermentation products from saccharolytic bacteria [59, 60]. In contrast, in the cecum, the acetate producing *Ruminiclostridium* spp. and *Ruminococcus gauvreauii* [61] within the family *Ruminococcaceae* processed large amounts of glycoside hydrolases and polysaccharide lyase [38]. These bacteria were negatively correlated with the dominated *Methanocorpusculum* spp., but positively correlated with *Saccharofermentans* spp. (Fig. 5b), which mainly produced succinate and lactate [62]. These results suggested that acetate and lactate possibly inhibited and proliferated the *Methanocorpusculum* spp. in the cecum, respectively. However, the basis for these correlations remains to be determined based on the metagenomic, metatranscriptomic analyses and methane production in future.

## Conclusions

In the present study, we provide additional insights into the development and correlation of bacteria and methanogens in the cecum and rumen of sika deer. The initially established bacterial community was significantly different from that at post weaning period. However, the established methanogens after birth (1 day) were persistently dominant in both the rumen and cecum of juvenile sika deer. These results documented that the different colonization event of bacteria and methanogens in GIT. We also found the heterogenetic distribution of methanogens between rumen and cecum, suggesting the role of the upper and lower GIT in affecting the methanogen community. However, the present study did not answer how the methanogens could establish very soon after birth. Future studies using metagenome and metatranscriptome will facilitate to understand this process and the heterogeneity in rumen and cecum. In summary, the manipulation strategies of the microbiota succession should take into account the different region of GIT regions.

## Methods

### Animals, management and diets

All animal-specific procedures were approved and authorized by the Chinese Academy of Agricultural Sciences Animal Care and Use Committee, and the Institute of Special Animal and Plant Sciences Wild Animal and Plant Subcommittee.

Fifteen neonatal sika deer (*Cervus nippon*) from our previous study were used in this study [63], which were authorized and obtained from the research farm of the Institute of Special Animal and Plant Sciences, Chinese Academy of Agricultural Sciences. Fifteen pairs of juvenile sika deer were kept with their dams in three pens, with 5 pairs of neonatal sika deer and dams in each pen. The juvenile sika deer suckled their young before weaning (60 days), also had access to the concentrate diets (64.5% corn, 19.7% soybean meal, 12.8% distiller dried grains with solubles and a 3% mixture of vitamins and mineral salts) and corn silage (concentrate: corn silage = 50:50, dry matter base). After weaning at day 60, five young animals were separated from their dams, and were maintained in an individual pen without any bedding materials, and were offered with forage and concentrate diets. All animals had free access to clean water during this study. Each five animals were euthanized by intravenous injection of barbituric acid (90 mg/kg body weight) and sacrificed on 1 day (Mean body weight = 5.31 ± 0.45 kg), 42 days (Mean body weight = 11.95 ± 1.23 kg) and 70 days (Mean body weight = 20.07 ± 1.75 kg), respectively. The rumen and cecum contents were collected, and preserved in liquid nitrogen prior to storage at − 80 °C until analysis. The other animal samples were preserved in the laboratory.

### Extracting genomic DNA, next generation sequencing and sequences analysis

Total genomic DNA was extracted from rumen and cecum contents using the QIAamp DNA Stool Mini Kit (QIAGEN, Valencia, CA, USA). The DNA samples was quantified using a Nanodrop 1000 spectrophotometer (Thermo Fisher Scientific, Wisconsin, USA), which were used to amplify the methanogen 16S rRNA gene using the archaea specific primers Ar915aF and Ar1386R in the rumen and cecum samples [64], and the bacterial 16S rRNA gene in rumen using the primers 341F and 806R [65] based on the previous application conditions. Each sample was amplified in triplicates. The resulting amplicons were purified using a QIAquick PCR Purification Kit (QIAGEN, Valencia, CA, USA), and then pooled in equimolar concentrations. The amplicon libraries were constructed by a NEBNext® Ultra™ DNA Library Prep Kit for Illumina (NEB, USA) according to the manufacturer’s instructions and index codes were added. The library quality was quantified on the Qubit® 2.0 Fluorometer (Thermo Scientific, Wisconsin, USA) and Agilent Bioanalyzer 2100 system. The amplicon library plus 5% PhiX control DNA was sequenced with the MiSeq 2 × 250 v2 kit (Illumina, San Diego, CA, USA) to generate paired 250-bp reads.

A total of 946,158 raw methanogen 16S rRNA gene sequences and a total of 477,021 raw bacteria 16S rRNA gene sequences were obtained. The following criteria were used to quality control: the minimum quality score was 25; the maximum number of errors in the barcode was 0; the allowed maximum length of homopolymer was 6; the number of mismatches in the primer was 0. The sequences with any ambiguous and unassigned characters were also removed. We obtained 723,689 sequences (methanogens) and 369,136 sequences (bacteria) for the further analysis using QIIME (Quantitative insights into microbial ecology) 1.9.0 software [66]. The sequences were clustered into operational taxonomic units (OTUs) using UPARSE [67], which were used to remove the chimera sequences [68], classify the taxonomy based on the RIM-DB for methanogens [69] and the SILVA database (version 125) for bacteria [70]. A phylogenetic tree was constructed using FastTree [71]. We sub-sampled the sequencing data of each sample to the lowest sequencing number after the remove of singletons. After that, the alpha diversity was subsequently calculated using QIIME 1.9.0 [66].

The principal coordinate analysis (PCoA) was applied to compare the methanogens (rumen and cecum) and bacteria (rumen) communities. Canonical correlation analysis (CCA) was conducted to identify the representative taxa at each time point from rumen and cecum [72]. R software (3.4.0) was applied to calculate the statistics analysis for OTU numbers, diversity indices and taxonomic abundance. Significance (*p* < 0.05) was based on the Benjamini-Hochberg corrected *p*-value from the Kruskal–Wallis test. All values were expressed as the mean unless otherwise stated.

### Co-occurrence between methanogens and bacteria in rumen and cecum

By using our previous data of cecum bacterial 16S rRNA gene [57], a co-occurrence network was constructed to examine the existence of correlations among the methanogens and bacteria across three time points [73]. Correlations have an absolute spearman’s correlation greater than 0.6 with a corrected significance level less than 0.05. Network were carried out with Cytoscape 3.5.1 using a force-directed algorithm [74].

## Additional files

Additional file 1: **Figure S1.** Bacterial community composition at phylum level in rumen of sika deer at 1 day, 42 days and 72 days. (PDF 154 kb)

Additional file 2: **Figure S2.** Comparing the methanogens in the rumen and cecum across three time points based on PCoA using the unweighted unifrac distance. (PDF 100 kb)

## Data Availability

The sequences in the present study were deposited in the SRA database under accession number SRP075175.

## Abbreviations

CCA: Canonical correlation analysis
GIT: Gastrointestinal tract
*Mbb.*: *Methanobrevibacter*
*Mmc.*: *Methanomassiliicoccaceae*
OTUs: Operational taxonomic units
PCoA: Principal coordinate analysis
QIIME: Quantitative insights into microbial ecology

## References

[1] Opio C, Gerber P, Mottet A, Falcucci A, Tempio G, MacLeod M, Vellinga T, Henderson B, Steinfeld H (2013). Greenhouse gas emissions from ruminant supply chains - A global life cycle assessment.

[2] Veneman JB, Saetnan ER, Clare AJ, Newbold CJ (2016). MitiGate; an online meta-analysis database for quantification of mitigation strategies for enteric methane emissions. Sci Total Environ.

[3] Tapio I, Snelling TJ, Strozzi F, Wallace RJ (2017). The ruminal microbiome associated with methane emissions from ruminant livestock. J Animal Sci Biotechnol.

[4] Hill J, McSweeney C, Wright ADG, Bishop-Hurley G, Kalantar-zadeh K (2016). Measuring methane production from ruminants. Trends Biotechnol.

[5] Wright ADG, Klieve AV (2011). Does the complexity of the rumen microbial ecology preclude methane mitigation?. Anim Feed Sci Technol.

[6] Borrel G, O’Toole PW, Harris HMB, Peyret P, Brugère J-F, Gribaldo S (2013). Phylogenomic data support a seventh order of methylotrophic methanogens and provide insights into the evolution of methanogenesis. Genome Biol Evol.

[7] Wolin MJ, Miller TL, Stewart CS, Hobson PN, Stewart CS (1997). Microbe-microbe interactions. The Rumen Microbial Ecosystem.

[8] Hungate RE (1967). Hydrogen as an intermediate in the rumen fermentation. Arch Mikrobiol.

[9] Johnson KA, Johnson DE (1995). Methane emissions from cattle. J Anim Sci.

[10] Yanez-Ruiz DR, Abecia L, Newbold CJ (2015). Manipulating rumen microbiome and fermentation through interventions during early life: a review. Front Microbiol.

[11] Malmuthuge N, Guan LL (2017). Understanding host-microbial interactions in rumen: searching the best opportunity for microbiota manipulation. J Anim Sci Biotechnol.

[12] Weimer PJ (2015). Redundancy, resilience, and host specificity of the ruminal microbiota: implications for engineering improved ruminal fermentations. Front Microbiol.

[13] Wang Z, Elekwachi CO, Jiao J, Wang M, Tang S, Zhou C (2017). Investigation and manipulation of metabolically active methanogen community composition during rumen development in black goats. Sci Rep.

[14] Jami E, Israel A, Kotser A, Mizrahi I (2013). Exploring the bovine rumen bacterial community from birth to adulthood. ISME J..

[15] Roehe R, Dewhurst RJ, Duthie C-A, Rooke JA, McKain N, Ross DW (2016). Bovine host genetic variation influences rumen microbial methane production with best selection criterion for low methane emitting and efficiently feed converting hosts based on metagenomic gene abundance. PLoS Genet.

[16] Difford GF, Plichta DR, Løvendahl P, Lassen J, Noel SJ, Højberg O (2018). Host genetics and the rumen microbiome jointly associate with methane emissions in dairy cows. PLoS Genet.

[17] Pérez-Barbería FJ (2017). Scaling methane emissions in ruminants and global estimates in wild populations. Sci Total Environ.

[18] Na Y, Li DH, Lee SR (2017). Effects of dietary forage-to-concentrate ratio on nutrient digestibility and enteric methane production in growing goats (*Capra hircus hircus*) and sika deer (*Cervus nippon hortulorum*). Asian-Australas J Anim Sci.

[19] Henderson G, Cox F, Ganesh S, Jonker A, Young W, Janssen PH (2015). Rumen microbial community composition varies with diet and host, but a core microbiome is found across a wide geographical range. Sci Rep.

[20] Siciliano-Jones J, Murphy MR (1989). Production of volatile fatty acids in the rumen and cecum-colon of steers as affected by forage: concentrate and forage physical form. J Dairy Sci.

[21] Murray RM, Bryant AM, Leng RA (1976). Rates of production of methane in the rumen and large intestine of sheep. Br J Nutr.

[22] Dill-McFarland KA, Breaker JD, Suen G (2017). Microbial succession in the gastrointestinal tract of dairy cows from 2 weeks to first lactation. Sci Rep.

[23] Zhou M, Chen Y, Griebel PJ, Guan LL (2014). Methanogen prevalence throughout the gastrointestinal tract of pre-weaned dairy calves. Gut Microbes.

[24] Guzman CE, Bereza-Malcolm LT, De Groef B, Franks AE (2015). Presence of selected methanogens, fibrolytic bacteria, and proteobacteria in the gastrointestinal tract of neonatal dairy calves from birth to 72 hours. PLoS One.

[25] Fonty G, Gouet P, Jouany J-P, Senaud J (1987). Establishment of the microflora and anaerobic fungi in the rumen of lambs. Microbiology..

[26] Li Z, Zhang Z, Xu C, Zhao J, Liu H, Fan Z (2014). Bacteria and methanogens differ along the gastrointestinal tract of Chinese roe deer (*Capreolus pygargus*). PLoS One.

[27] Lyu Z, Lu Y (2017). Metabolic shift at the class level sheds light on adaptation of methanogens to oxidative environments. ISME J.

[28] Gilmore SP, Henske JK, Sexton JA, Solomon KV, Seppälä S, Yoo JI (2017). Genomic analysis of methanogenic archaea reveals a shift towards energy conservation. BMC Genomics.

[29] Salgado-Flores A, Hagen LH, Ishaq SL, Zamanzadeh M, Wright A-DG, Pope PB (2016). Rumen and cecum microbiomes in reindeer (*Rangifer tarandus tarandus*) are changed in response to a lichen diet and may affect enteric methane emissions. PLoS One.

[30] Rey M, Enjalbert F, Combes S, Cauquil L, Bouchez O, Monteils V (2014). Establishment of ruminal bacterial community in dairy calves from birth to weaning is sequential. J Appl Microbiol.

[31] Dias J, Marcondes MI, Noronha MF, Resende RT, Machado FS, Mantovani HC (2017). Effect of pre-weaning diet on the ruminal archaeal, bacterial, and fungal communities of dairy calves. Front Microbiol.

[32] Patel V, Patel AK, Parmar NR, Patel AB, Reddy B, Joshi CG (2014). Characterization of the rumen microbiome of Indian Kankrej cattle (*Bos indicus*) adapted to different forage diet. Appl Microbiol Biotechnol.

[33] Singh KM, Patel AK, Shah RK, Reddy B, Joshi CG (2015). Potential functional gene diversity involved in methanogenesis and methanogenic community structure in Indian buffalo (*Bubalus bubalis*) rumen. J Appl Genet.

[34] Malmuthuge N, Griebel PJ, Guan LL (2014). Taxonomic identification of commensal bacteria associated with the mucosa and digesta throughout the gastrointestinal tracts of preweaned calves. Appl Environ Microbiol.

[35] Wang Z, Elekwachi C, Jiao J, Wang M, Tang S, Zhou C (2017). Changes in metabolically active bacterial community during rumen development, and their alteration by rhubarb root powder revealed by 16S rRNA amplicon sequencing. Front Microbiol.

[36] Mueller NT, Bakacs E, Combellick J, Grigoryan Z, Dominguez-Bello MG (2015). The infant microbiome development: mom matters. Trends Mol Med.

[37] Kalita A, Hu J, Torres AG (2014). Recent advances in adherence and invasion of pathogenic Escherichia coli. Curr Opin Infect Dis.

[38] Seshadri R, Leahy SC, Attwood GT, Teh KH, Lambie SC, Cookson AL (2018). Cultivation and sequencing of rumen microbiome members from the Hungate1000 collection. Nat Biotechnol.

[39] Purushe J, Fouts D, Morrison M, White B, Mackie R, Coutinho P (2010). Comparative genome analysis of Prevotella ruminicola and *Prevotella bryantii*: insights into their environmental niche. Microb Ecol.

[40] Flint HJ, Bayer EA, Rincon MT, Lamed R, White BA (2008). Polysaccharide utilization by gut bacteria: potential for new insights from genomic analysis. Nat Rev Microbiol.

[41] Béra-Maillet C, Ribot Y, Forano E (2004). Fiber-degrading systems of different strains of the genus *Fibrobacter*. Appl Environ Microbiol.

[42] Li RW, Connor EE, Li CJ, Baldwin RL, Sparks ME (2012). Characterization of the rumen microbiota of pre-ruminant calves using metagenomic tools. Environ Microbiol.

[43] Skillman LC, Evans PN, Naylor GE, Morvan B, Jarvis GN, Joblin KN (2004). 16S ribosomal DNA-directed PCR primers for ruminal methanogens and identification of methanogens colonising young lambs. Anaerobe..

[44] McDougall EI (1948). Studies on ruminant saliva. 1. The composition and output of sheep's saliva. Biochem J.

[45] Huhtanen P, Ahvenjärvi S, Broderick GA, Reynal SM, Shingfield KJ (2010). Quantifying ruminal digestion of organic matter and neutral detergent fiber using the omasal sampling technique in cattle-a meta-analysis1. J Dairy Sci.

[46] Moharrery A, Larsen M, Weisbjerg MR (2014). Starch digestion in the rumen, small intestine, and hind gut of dairy cows - a meta-analysis. Anim Feed Sci Technol.

[47] Popova M, Morgavi DP, Martin C (2013). Methanogens and methanogenesis in the rumens and ceca of lambs fed two different high-grain-content diets. Appl Environ Microbiol.

[48] Popova M, McGovern E, McCabe MS, Martin C, Doreau M, Arbre M (2017). The structural and functional capacity of ruminal and cecal microbiota in growing cattle was unaffected by dietary supplementation of linseed oil and nitrate. Front Microbiol.

[49] Ohkuma M, Noda S, Horikoshi K, Kudo T (1995). Phylogeny of symbiotic methanogens in the gut of the termite Reticulitermes speratus. FEMS Microbiol Lett.

[50] Luo YH, Wright ADG, Li YL, Li H, Yang QH, Luo LJ (2013). Diversity of methanogens in the hindgut of captive white rhinoceroses, *Ceratotherium simum*. BMC Microbiol.

[51] Fernandes KA, Kittelmann S, Rogers CW, Gee EK, Bolwell CF, Bermingham EN (2014). Faecal microbiota of forage-fed horses in New Zealand and the population dynamics of microbial communities following dietary change. PLoS One.

[52] Anderson IJ, Sieprawska-Lupa M, Goltsman E, Lapidus A, Copeland A, Glavina Del Rio T (2009). Complete genome sequence of *Methanocorpusculum labreanum* type strain Z. Stand Genomic Sci.

[53] Bapteste É, Brochier C, Boucher Y (2005). Higher-level classification of the archaea: evolution of methanogenesis and methanogens. Archaea..

[54] Poulsen M, Schwab C, Jensen BB, Engberg RM, Spang A, Canibe N (2013). Methylotrophic methanogenic *Thermoplasmata* implicated in reduced methane emissions from bovine rumen. Nat Commun.

[55] Borrel G, Harris HMB, Tottey W, Mihajlovski A, Parisot N, Peyretaillade E (2012). Genome sequence of “Candidatus *Methanomethylophilus alvus*” Mx1201, a methanogenic archaeon from the human gut belonging to a seventh order of methanogens. J Bacteriol.

[56] Borrel G, McCann A, Deane J, Neto MC, Lynch DB, Brugère J-F (2017). Genomics and metagenomics of trimethylamine-utilizing archaea in the human gut microbiome. ISME J..

[57] Li G, Si H, Zhang T, Nan W, Wang X, Li Z (2019). Bacterial community and metabolome shifts in the cecum and colon of captive sika deer (*Cervus nippon*) from birth to post weaning. FEMS Microbiol Lett.

[58] Rosero JA, Killer JÍ, Sechovcová H, Mrázek J, Benada OI, Fliegerová KI (2016). Reclassification of Eubacterium rectale (Hauduroy et al. 1937) Prévot 1938 in a new genus *Agathobacter* gen. nov. as *Agathobacter rectalis* comb. nov., and description of *Agathobacter ruminis* sp. nov., isolated from the rumen contents of sheep and cows. Int J Syst Evol Microbiol.

[59] Samuel BS, Hansen EE, Manchester JK, Coutinho PM, Henrissat B, Fulton R (2007). Genomic and metabolic adaptations of *Methanobrevibacter smithii* to the human gut. Proc Natl Acad Sci U S A.

[60] Pozuelo M, Panda S, Santiago A, Mendez S, Accarino A, Santos J (2015). Reduction of butyrate- and methane-producing microorganisms in patients with irritable bowel syndrome. Sci Rep.

[61] Domingo M-C, Huletsky A, Boissinot M, Bernard KA, Picard FJ, Bergeron MG (2008). *Ruminococcus gauvreauii* sp. nov., a glycopeptide-resistant species isolated from a human faecal specimen. Int J Syst Evol Microbiol.

[62] Shuangya C: Saccharofermentans. In: Bergey’s Manual of Systematics of Archaea and Bacteria. Edited by W. B. Whitman FR, P. Kämpfer, M. Trujillo, J. Chun, P. DeVos, B. Hedlund, S. Dedysh; 2017.

[63] Li Z, Wang X, Zhang T, Si H, Nan W, Xu C (2018). The development of microbiota and metabolome in small intestine of sika deer (*Cervus nippon*) from birth to weaning. Front Microbiol.

[64] Kittelmann S, Seedorf H, Walters WA, Clemente JC, Knight R, Gordon JI (2013). Simultaneous amplicon sequencing to explore co-occurrence patterns of bacterial, archaeal and eukaryotic microorganisms in rumen microbial communities. PLoS One.

[65] Klindworth A, Pruesse E, Schweer T, Peplies J, Quast C, Horn M (2012). Evaluation of general 16S ribosomal RNA gene PCR primers for classical and next-generation sequencing-based diversity studies. Nucleic Acids Res.

[66] Caporaso JG, Kuczynski J, Stombaugh J, Bittinger K, Bushman FD, Costello EK (2010). QIIME allows analysis of high-throughput community sequencing data. Nat Meth..

[67] Edgar RC (2013). UPARSE: highly accurate OTU sequences from microbial amplicon reads. Nat Meth.

[68] Edgar RC, Haas BJ, Clemente JC, Quince C, Knight R (2011). UCHIME improves sensitivity and speed of chimera detection. Bioinformatics..

[69] Seedorf H, Kittelmann S, Henderson G, Janssen PH (2014). RIM-DB: a taxonomic framework for community structure analysis of methanogenic archaea from the rumen and other intestinal environments. PeerJ..

[70] Quast C, Pruesse E, Yilmaz P, Gerken J, Schweer T, Yarza P (2013). The SILVA ribosomal RNA gene database project: improved data processing and web-based tools. Nucleic Acids Res.

[71] Price MN, Dehal PS, Arkin AP (2009). FastTree: computing large minimum evolution trees with profiles instead of a distance matrix. Mol Biol Evol.

[72] Dufrene M, Legendre P (1997). Species assemblages and indicator species: the need for a flexible asymmetrical approach. Ecol Monogr.

[73] Li Zhipeng, Wright André-Denis G., Liu Hanlu, Fan Zhongyuan, Yang Fuhe, Zhang Zhigang, Li Guangyu (2015). Response of the Rumen Microbiota of Sika Deer (Cervus nippon) Fed Different Concentrations of Tannin Rich Plants. PLOS ONE.

[74] Smoot ME, Ono K, Ruscheinski J, Wang PL, Ideker T (2011). Cytoscape 2.8: new features for data integration and network visualization. Bioinformatics..

