# Supplementary figures and images for: Heterogeneous development of methanogens and the correlation with bacteria in the rumen and cecum of sika deer (Cervus nippon) during early life suggest different ecology relevance
Source: BMC Microbiol. 2019 Jun 11;19:129. doi: 10.1186/s12866-019-1504-9 (PMC6560721; doi:10.1186/s12866-019-1504-9)

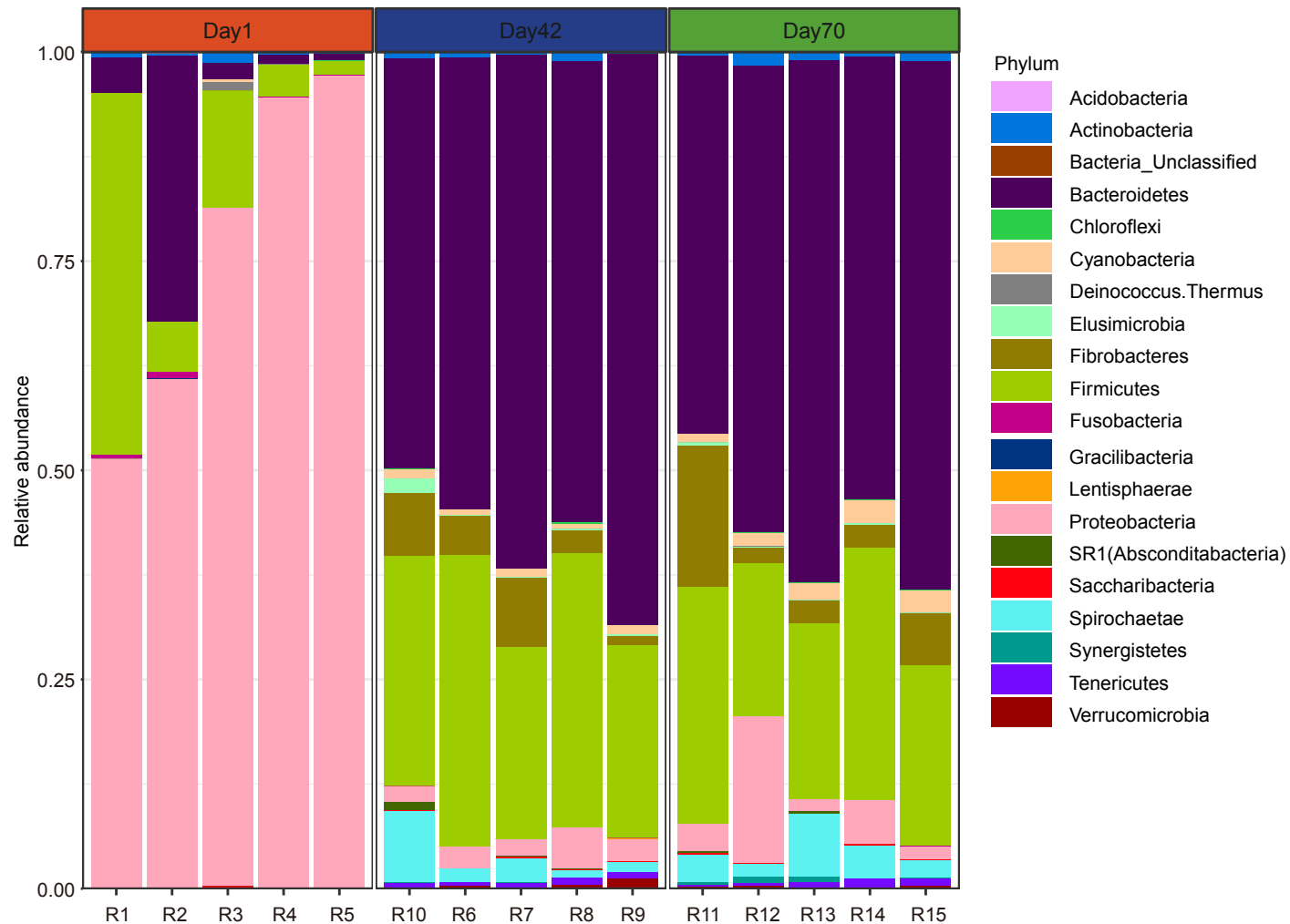

Supplement: Supplementary file 1 — Figure S1. Bacterial community composition at phylum level in rumen of sika deer at 1 day, 42 days and 72 days. (PDF 154 kb) [file 12866_2019_1504_MOESM1_ESM.pdf]

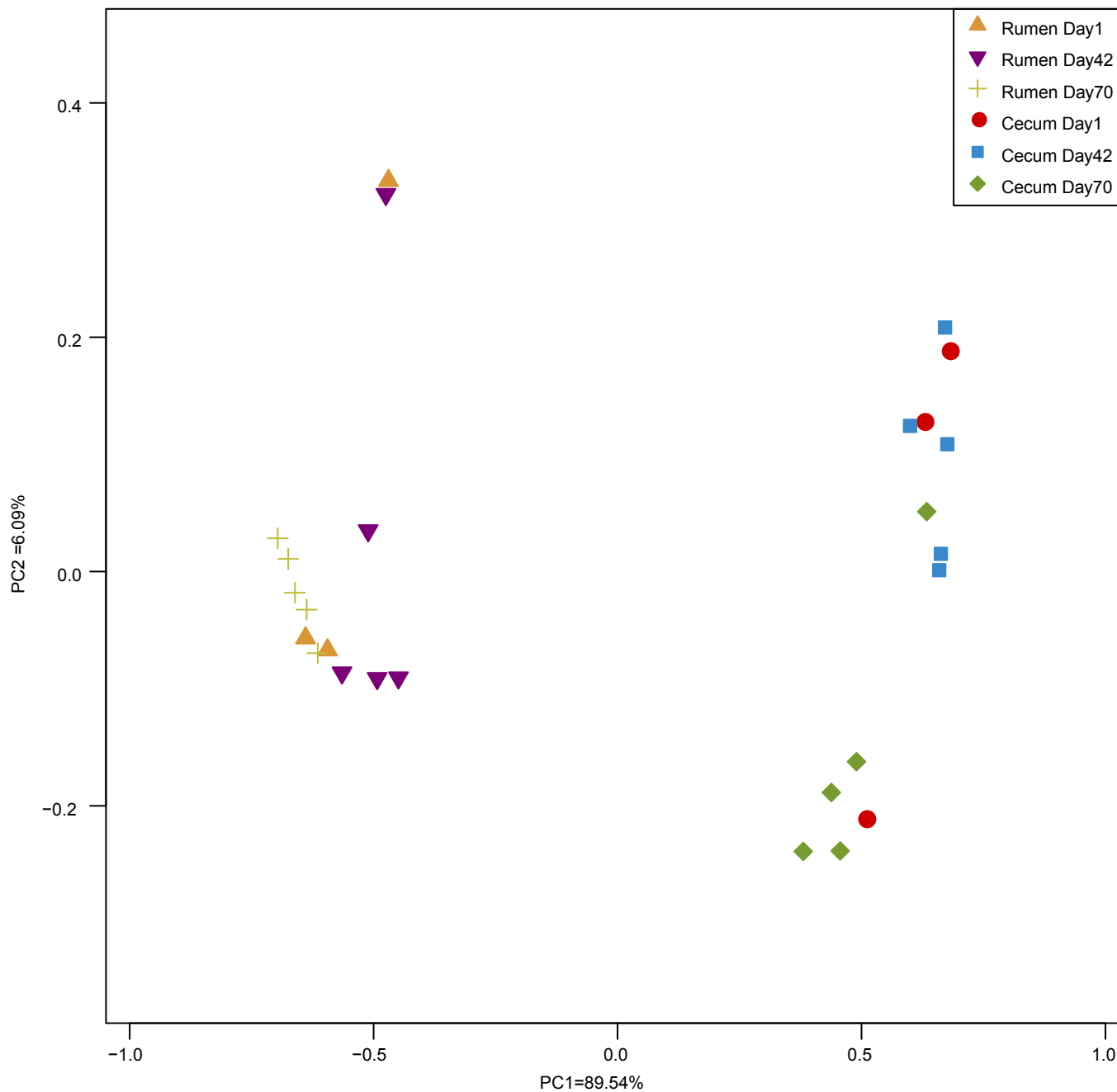

Supplement: Supplementary file 2 — Figure S2. Comparing the methanogens in the rumen and cecum across three time points based on PCoA using the unweighted unifrac distance. (PDF 100 kb) [file 12866_2019_1504_MOESM2_ESM.pdf]
